# Landscape of H5 Infections in ASEAN Region: Past Insights, Present Realities, & Future Strategies

**DOI:** 10.3390/v17040535

**Published:** 2025-04-06

**Authors:** Muhammad Nur Adam Hatta, Yi Xin Nga, Ezryn Najwa Amirnuddin, Siti Nuraisyah Muzafar, Jasmine Elanie Khairat

**Affiliations:** 1 Institute of Biological Sciences, Faculty of Science, Universiti Malaya, Kuala Lumpur 50603, Malaysia; adamhatta@um.edu.my (M.N.A.H.); u2002129@siswa.um.edu.my (E.N.A.); u2001505@siswa.um.edu.my (S.N.M.); 2 School of Biological Sciences, Faculty of Biology, Medicine and Health, The University of Manchester, Manchester M13 9PL, UK; yi.nga@student.manchester.ac.uk; 3 Center for Natural Products & Drug Research, Universiti Malaya, Kuala Lumpur 50603, Malaysia

**Keywords:** H5, infection, ASEAN, epidemiology, socioeconomics, regional responses, zoonotic, One Health

## Abstract

The H5 Avian Influenza A virus infection has emerged as a global concern, particularly in the ASEAN region. This viral infection poses a significant threat to the poultry industry, public health, and regional economies. This region’s reliance on poultry production and the zoonotic potential of H5 subtypes, with documented transmission to various mammalian species and humans, necessitates proactive mitigation strategies. Over the years, comprehensive efforts such as surveillance, vaccination programs, biosecurity measures, and public health education have been implemented to keep outbreaks at bay. In this review, we provide a thorough overview of the H5 infections in the ASEAN region, focusing on the unique challenges and successes in this geographic area. We analyze epidemiological trends, including specific high-risk populations and transmission patterns, and assess the socioeconomic impact of H5 outbreaks on local communities. We also examine regional responses, highlighting innovative surveillance programs, vaccination strategies, and biosecurity measures implemented to control the virus. Furthermore, we explore the crucial role of the One Health approach, emphasizing interdisciplinary collaboration between human, animal, and environmental health sectors. Finally, we discuss future strategies for prevention and control, including the importance of regional cooperation in combating this evolving threat. Through this, we aim to provide valuable insights to the public, policymakers, and researchers involved in tackling H5 infections globally.

## 1. Introduction

**Figure 1:**
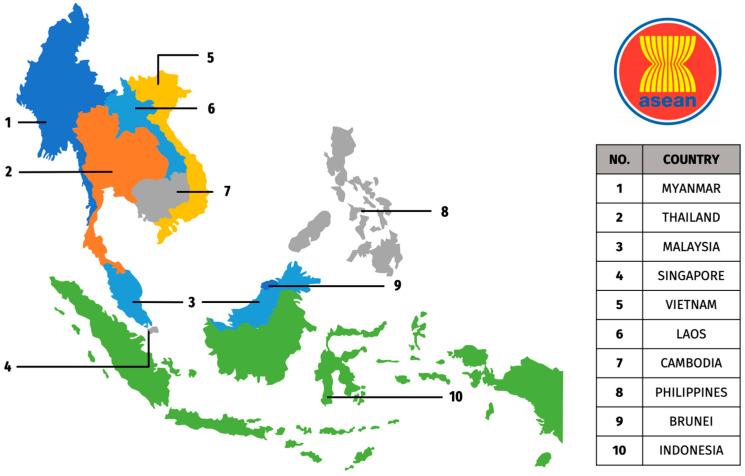
ASEAN member states and their geographical locations.

The first emergence of human Influenza A virus (IAV) H5 infection was reported in 1997 through an outbreak in Hong Kong [1]. Initially, H5 infections were found mainly in poultry animals. However, through natural genetic reassortment, zoonotic transmission to humans has become possible, thus triggering the alarm of the next potential pandemic. The advancement of technology and research throughout the years resulted in the identification of more H5 strains worldwide, especially in ASEAN (Association of Southeast Asian Nations) countries (Figure 1). This region has been categorized as a hotspot for pandemic potential emerging infectious diseases [2]. Since 2003, countries such as Cambodia, Indonesia, Laos, Malaysia, Myanmar, Thailand, and Vietnam have been affected by the Highly Pathogenic Avian Influenza (HPAI)—H5N1 subtype, leading to significant economic losses in the poultry sector and posing a continued threat to public health [3]. Outbreaks of diverse subtypes of H5 IAVs, particularly H5N1, H5N2, H5N6, and H5N8, have been reported throughout the years in the ASEAN region.

The ability of H5 IAVs to infect multiple hosts will eventually affect the complex dynamic systems of a region in terms of their biology, society, ecology, and technology [4]. Progressively, this will affect the agricultural economy, which is one of the important sectors in most of the ASEAN countries. Outbreaks can lead to widespread culling of poultry, trade restrictions, and loss of consumer confidence, jeopardizing biosecurity efforts and raising public health concerns. A recent study has identified the presence of H5 IAVs in wild animal and dairy farms, indicating multidirectional transmission between various species, which raises concerns about the virus’s ability to adapt and spread [5,6]. This virus transfer ability causes disturbance of the normal biodiversity, possibly interfering with the food chain in the future. In this review, we briefly discussed the emergence and importance of the predominant H5 subtype in the ASEAN region. This study also examines past and present approaches for controlling H5 IAVs and proposes future actions. By analyzing these strategies, we hope to provide a fundamental understanding of H5 IAVs eradication efforts.

## 2. Epidemiology, Transmission, and Zoonotic Potential of H5 Infections in the ASEAN Region

Briefly, all IAVs are made up of eight segmented genes that encode 10 important proteins for the viral life cycle that include PB1 (polymerase basic 1), PB2 (polymerase basic 2), PA (polymerase acidic protein), HA (hemagglutinin), NA (neuraminidase), M1 (matrix 1 protein), M2 (matrix 2 protein), NS1 (nonstructural protein 1), NEP (nuclear export protein), and NP (nucleoprotein) [7,8]. Two viral glycoproteins, HA and NA, are the common targets for genetic variation [9]. There are 18 HA and 11 NA subtypes for IAVs known worldwide [10]. In addition to defining the IAV subtype, HA and NA genes facilitate the interaction between IAVs and host cells and their ability to spread. The H5 subtype shares common features with other IAV subtypes. As of November 2024, there are at least nine H5 subtypes that have been identified worldwide [11]. Nonetheless, only four predominant H5 subtypes have been reported circulating in the ASEAN and/or Asian region: H5N1, H5N2, H5N6, and H5N8 [12].

Most of these subtypes successfully infect avian species, such as chickens and ducks, with a high mortality rate. Studies show that these subtypes’ infection in poultry animals is able to cause a mortality rate of 70–100% in a very short incubation period [13,14,15,16,17]. On the other hand, these infections will be highly contagious to the other birds in the same flocks via direct contact, contaminated environment, and/or aerosol transmission [18]. It has been shown that most IAVs are able to survive on contaminated surfaces for more than 24 h, increasing the transmission rate [19]. Brief epidemiology, transmission, and evidence of zoonotic potential on these selected H5 subtypes are further discussed in this section.

### 2.1. H5N1 Subtype

The HPAI H5N1 subtype has been a significant public health concern in the ASEAN region since its discovery in the late 1990s. This subtype was first isolated from domestic geese in the Guangdong Province of China in 1996 (A/Goose/Guangdong/1/96) [20], followed by an outbreak in the subsequent year in Hong Kong [21]. After a temporary hiatus, a big wave of H5N1 infection re-emerged in 2003 and rapidly affected at least seven Asian countries, including Cambodia, China, Indonesia, Japan, Laos, Thailand, and Vietnam [3]. Over time, the virus spread to many other parts of Asia, Europe, and Africa [22], predominantly by bird migration and poultry transport activity [23], and finally reached America in 2021 [24]. Although aquatic bird species serve as primary reservoirs for the H5N1 virus, the virus also could infect a diverse spectrum of hosts, including wild birds, domestic poultry, and marine mammals [22]. In the ASEAN region, H5N1 outbreaks are often seasonal, with a notable increase in cases during the cooler months from January to March. These outbreaks frequently coincide with seasonal flu, raising concerns about co-infections and potential viral gene reassortments, which may result in the emergence of new strains capable of human-to-human transmission [25].

The first known transmission of H5N1 to humans happened in Hong Kong in 1997, where it resulted in clusters of 18 cases, six of which were fatal [22]. This outbreak caused approximately 1.5 million poultry in Hong Kong farms and markets to be slaughtered to prevent the situation from worsening [26]. The virus has demonstrated its zoonotic potential, with human infections occurring through direct contact with infected poultry, via contaminated surfaces or virus in the air in droplet form [11]. The US CDC also reported 29 sporadic H5N1 human infections globally from January 2022 to June 2024 with at least seven deaths and 15 critical illnesses. The outbreaks were primarily associated with live poultry markets, where the high density and co-mingling of different bird species created optimal conditions for viral reassortment and transmission [21]. The continuous interaction between poultry and wild birds has led to the evolution and diversification of H5N1 into numerous phylogenetic clades [22]. Notably, clade 2.3.4.4b viruses undergo genetic reassortment by acquiring gene segments from other influenza viruses, giving rise to H5N2, H5N5, H5N6, and H5N8 subtypes [12,27]. Control measures for prevention and response to H5N1 outbreaks, such as closure of these markets, mass culling campaigns, and poultry vaccination using inactivated H5N1, had been implemented. Despite these efforts, H5N1 has continued to circulate in domestic and wild bird populations, resulting in sporadic human cases with increasing fatality rates since its re-emergence in 2003 [3,12]. Fortunately, no sustained human-to-human transmission has ever been reported [22,26,28]. However, the virus’s potential to cross the species barrier poses a significant threat, particularly in regions where poultry farming is a major economic activity.

### 2.2. H5N2 Subtype

Since the 1980s, the emergence of the H5N2 subtype has been reported mainly in Western countries [29]. This subtype could be categorized under both LPAI (Low Pathogenic Avian Influenza) and HPAI groups based on the isolated strains during any outbreaks. Interestingly, this dual-group category resulted in the varied pathogenicity causing difficulty for infection control. LPAI H5N2 infection symptoms are often mild with a lower mortality rate compared to HPAI cases that have a high mortality rate with multiple clinical manifestations such as lethargy, paralysis, neurological complications, and severe respiratory issues. In Asia, the first outbreak of H5N2 was first reported in Taiwan in 2003 [30]. This resulted in 21 chicken farms being culled to avoid widespread viral transmission [31]. Later in 2005, Japan reported their first case of H5N2 in multiple chicken farms in Ibaraki and Saitama province, causing approximately 5.7 million birds to be killed [32]. Subsequently, this 2005 outbreak in Japan resulted in the first known human H5N2 cases through serological testing on the affected farmers [33].

Fast forward to 2024, Mexico has reported another laboratory-confirmed case of H5N2 infection in humans followed by mortality [34]. This subtype was once categorized as a low threat to humans but now has come to show its true potential. In the ASEAN region, no known H5N2 outbreak was ever reported. However, this subtype has been detected during isolated surveillance in 2004 and 2014 in Malaysia [35]. Other than humans, interspecies transmissibility of H5N2 was detected in Korea, isolated from pigs, which are a great intermediate host, especially in causing genetic shift, raising the risk of new strains emerging with unpredictable transmissibility [36]. Most of the H5N2 cases in the Asia region are highly associated with the American lineage, raising concerns about transcontinental transmission of the virus [37]. Migratory birds became the main culprit as H5N2 was present during surveillance conducted in Japan and South Korea [38,39]. To add, an isolated case of H5N2 in dogs was reported in China back in 2009 as a result of complex genetic reassortment [40].

### 2.3. H5N6 Subtype

The H5N6 subtype was first identified in domestic poultry in Sichuan Province, China, in 2014, and subsequently emerged in the ASEAN region of Vietnam and Laos in 2014 [41,42]. From 2014 to 2022, China experienced at least 72 H5N6 outbreaks, resulting in the culling of approximately 230,000 birds [43]. Based on phylogenetic analysis, the H5N6 subtype is considered a novel genetic reassortment between H5N1 and H6N6 viruses, which had been commonly circulating among poultry in China, and had evolved into 2 distinct lineages (Sichuan and Jiangxi) [44,45]. Notably, the H5N6 strain found in Vietnam was isolated from dead quails during influenza outbreaks [12]. The migration of wild birds between wintering and breeding sites has played a crucial role in the global spread of H5N6 influenza viruses. During the 2017 breeding season and the following winter, the genetic diversity of H5N6 viruses increased significantly, facilitating their dissemination to multiple Asian (China, Bangladesh, South Korea, Japan) and European countries (Greece, England, Switzerland, Italy, the Netherlands, Germany, Denmark, etc.) [46].

A comparative analysis revealed that H5N6 infection typically causes mild illness in natural hosts like ducks, but it can be severe and even fatal in spillover hosts such as chickens and humans [47,48]. In April 2014, the first fatal case of H5N6 infection in humans was reported in Sichuan, China [45]. The virus later spread beyond China, with the first human case outside of China reported in Laos in 2021 [12]. Since 2014, a total of 92 human H5N6 infections, including 37 deaths, have been reported to the WHO in the Western Pacific Region [49]. While certain molecular markers in H5N6 suggest potential adaptation to mammals [50], a study on ferrets has shown that the virus does not efficiently adapt to intraspecies transmission [51]. Nonetheless, H5N6 has demonstrated its zoonotic potential, meaning that while human transmission is currently inefficient, it could still potentially cause a human pandemic.

### 2.4. H5N8 Subtype

One of the earliest reported H5N8 outbreaks occurred in 1983 on a poultry farm in Ireland, affecting turkeys, chickens, and ducks [52]. A recent study indicates that H5N8 may have a high mortality rate, reaching up to 70% with varied clinical manifestations [13]. While H5N8 outbreaks have been reported worldwide, no known cases have been identified in the ASEAN region. However, the emergence of H5N8 in neighboring Asian countries, including South Korea, China, India, Taiwan, and Japan, increases the risk of its introduction to Southeast Asia [53,54,55,56]. Most H5N8 infections are caused by subclade 2.3.4.4b, which arose from the genetic evolution of H5N1 subclade 2.3.4 variants [55]. In 2014, an H5N8 subclade 2.3.4.4b outbreak in South Korea led to the culling of approximately 12 million ducks [57]. Fast forward to December 2016, further H5N8 outbreaks across the region resulted in the culling of over 18 million birds in South Korea and 800,000 birds in Japan.

Apart from commercialized poultry animal infections, the H5N8 subtype has also been discovered in wild migratory birds [58]. Studies indicate that these migratory birds contribute to the transcontinental spread of the virus through fecal deposition and the transport of infected carcasses [59]. This poses a heightened risk of transmission to the ASEAN region, particularly due to migratory bird movements in Asian countries with reported H5N8 outbreaks. Most H5 HPAI subtypes, including H5N8, have the potential to cross the species barrier. One study reported that H5N8-infected birds cause a successful interspecies transmission to seals and foxes, causing neurological complications [60,61]. In December 2020, the first case of H5N8 bird-to-human transmission was reported in Astrakhan, Russia, affecting seven poultry farm workers who remained asymptomatic [62]. While prolonged surveillance of these infected individuals revealed no evidence of human-to-human transmission, this event demonstrates the virus’s capacity to infect multiple host species.

## 3. Socioeconomic Impacts

HPAI outbreaks, particularly those caused by H5 subtypes, have had profound socioeconomic consequences on affected countries, particularly in ASEAN nations where poultry production is a vital livelihood. The necessity to cull infected and at-risk birds during outbreaks has led to substantial financial losses for farmers and related industries, disrupting local economies, particularly in rural areas where poultry farming is a primary source of income [63,64]. For example, during the 2003–2004 H5N1 outbreaks, Vietnam and Thailand experienced the highest losses, with 15–18% of their poultry populations culled [65]. In Vietnam, this resulted in a 15% decline in poultry production and economic losses equivalent to approximately 0.1% of GDP (USD 45 million) [66]. The Indonesian poultry industry also suffered significant disruption, with an estimated 11 million chickens culled between 2003 and 2009 [67]. Furthermore, H5 outbreaks prompted the imposition of import bans on poultry products from disease-affected countries, causing supply shortages, price increases, and, eventually, trade disruptions. Restrictions on exports from Asian countries during the 2004–2005 H5N1 outbreaks contributed to an approximate 20% increase in international poultry prices [65]. In 2004, Southeast Asia’s economies faced a significant impact, resulting in an 8% decline in global poultry trade and a dramatic 36.8% decrease in intra-regional trade [68].

Beyond the direct impact on agriculture, H5 influenza outbreaks have far-reaching socioeconomic consequences. These outbreaks place a significant burden on the healthcare systems, requiring increased resources for treatment, prevention measures, public health interventions, and subsidies for small farmers [67,69]. Additionally, outbreaks can lead to substantial productivity losses due to illness-related absenteeism and disruptions in transportation and supply chains. For instance, the impact of influenza pneumonia in Thailand between September 2003 and August 2004 shows that lost productivity accounted for a staggering 50–53% of the total economic losses, which ranged from USD 24 million to 63 million. Transportation disruptions added another 3–7%, while direct medical expenses constituted 43% of the overall financial burden [70,71]. Furthermore, outbreaks also raised food safety concerns, eroding consumers’ confidence in poultry products, leading to decreased consumption and further economic strain on the poultry industry. Many Thai consumers opted for alternative protein sources, which exacerbated the financial strain faced by the poultry industry at that time [72,73]. In summary, H5 influenza outbreaks have profound socioeconomic repercussions that extend beyond the immediate impact on agricultural production. These outbreaks disrupt local economies, international trade, healthcare systems, and consumer confidence, posing significant challenges for both affected communities and governments in managing the direct and indirect consequences.

## 4. ASEAN Regional Responses and Collaborative Efforts

**Figure 2:**
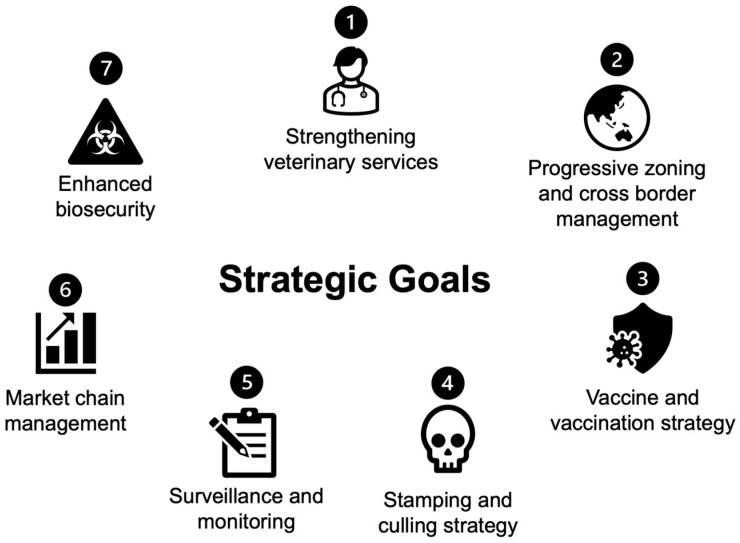
Seven main strategic goals for pre- and post-2020 ASEAN framework for HPAI management.

To address the threat of H5 avian influenza and its socioeconomic impact, ASEAN member states have collaborated on a regional avian influenza virus response framework since 2003. This framework has evolved through several iterations, including the establishment of the ASEAN HPAI Taskforce in 2004, the Regional Framework for the Control and Eradication of HPAI in ASEAN in 2005, the Regional Strategy 2008–2010, and the Roadmap for an HPAI-free ASEAN Community by 2020. In 2023, the 45th meeting of the ASEAN Ministers on Agriculture and Forestry led to the adoption of the Post-2020 Avian Influenza Control Framework [12]. This updated framework prioritizes the eradication of HPAI in domestic poultry, strengthens the surveillance of human transmission, and addresses interconnected challenges related to public health, food security, and emerging new diseases. It outlines seven strategic goals focused on the prevention, control, and eradication efforts (Figure 2).

### 4.1. Strengthening Veterinary Services

Recognizing the critical role of veterinary services in combating HPAI outbreaks, the framework calls for enhanced capacity building, clearer guidelines for disease control, and the implementation of animal health legislation [74]. This will provide the risk mitigation of the diseases along the livestock value chain. This includes establishing a clear job scope and responsibilities for veterinary professionals and promoting collaboration with other sectors, ensuring proper HPAI controls and prevention as implemented by Indonesia [75].

### 4.2. Progressive Zoning and Cross-Border Management

In this aspect, the framework advocates for a risk-based zoning system to manage the movement of poultry products and prevent disease spread. For instance, poultry products from this zoning area need a certification system that represents the AIV-free status even at the border, as practiced by the Singapore authorities [76]. This ensures the accessibility of health information across borders. ASEAN member countries have taken control of this management system by implementing the standardized Good Animal Husbandry Practices (GAHP) as part of its food safety plans for emerging infectious diseases. From a case study in Indonesia, an improvement in local farm management was observed, driven by the successful execution of the ASEAN-GAHP [77]. These approaches allow targeted and scalable responses for AIV disease management, particularly H5.

### 4.3. Vaccine and Vaccination Strategy

The framework stressed the importance of accessible, safe, and effective H5 vaccines for disease control. This effort requires different organizations, institutes, vaccine manufacturers, and local governments to work together to ensure sufficient vaccine production and distribution. This strategy will strongly affect the overall control measure in managing H5 outbreaks [78]. Promising vaccine candidates, such as inactivated trivalent vaccines, have demonstrated efficacy in protecting various poultry species [79]. One case study on the Vietnam clade 2.3.2.1a H5N1 showed that both single and double homologous vaccines protected adult-layer chickens, safeguarding egg production [80]. Additionally, some ASEAN countries, including Thailand, Singapore, Laos, and the Philippines, have implemented programs to provide free influenza vaccines to high-risk individuals, aiming to prevent zoonotic transmission and protect public health [71].

### 4.4. Stamping Out and Culling

Another common strategic control that has been implemented includes the depopulation of AIV-infected or at-risk poultry flocks. Contaminated areas and materials will be cleaned and disinfected with proper agents to avoid cross-contamination. This approach has been used to control different types of virus infections, such as Nipah virus, Marburg virus, and African Swine Fever virus [81,82,83]. These measures, while effective in eliminating the virus from affected populations, can result in significant financial loss for farmers. To mitigate this impact, compensation schemes have been implemented in several ASEAN countries, including Malaysia, Thailand, and Vietnam, providing affected farmers with 50–100% of the market value for culled birds. Furthermore, targeted zoning approaches involving stamping out and culling in designated areas have been adopted to limit the economic impact and prevent further disease spread [84,85,86].

### 4.5. Surveillance and Monitoring

The framework emphasizes the importance of collaborative efforts across the ASEAN region to gather comprehensive data on AIV emergence and transmission. This includes continuous monitoring of poultry populations, wild birds, and human cases. Surveillance programs need to be carried out throughout the years with systematic actions. The framework encourages the integration of national surveillance programs with the WHO’s Global Influenza Surveillance and Response System (GISRS) to enhance data sharing and global coordination [87]. Consequently, this benefits the early detection of outbreaks and control programs, where a holistic monitoring program requires coordination between government and private agencies throughout the ASEAN area. Examples of successful surveillance initiatives within ASEAN include Myanmar’s annual sero-surveillance of H5 in ducks, providing substantial information regarding potential outbreaks [88]. Similarly in Malaysia, university researchers are conducting surveillance by developing models of influenza infection to predict and track the spread of the virus [89].

### 4.6. Market Chain Management

Managing AIV outbreaks within the market chain involves a complex set of measured aims. This includes how the product moves from poultry farms to the end consumer, as well as mitigating the overall economic impact of the poultry industry. This requires a comprehensive approach that integrates various strategies, including surveillance, movement controls, zoning, biosecurity, and vaccination strategies, ensuring efficient controls of potential outbreaks. Of particular importance is the implementation of good biosafety practices in live bird markets (LBM), which can serve as potential hubs for virus transmission [90]. This effort was found to be effective in controlling AIV outbreaks, especially in smaller LBMs [91]. For example, in 2008, Cambodia established a network for monitoring poultry movements and conducting surveillance in LBMs to identify and mitigate potential H5 hotspots [92].

### 4.7. Enhanced Biosecurity

Implementation of strict biosecurity practices is needed to ensure effective AIV controls. This requires implementing comprehensive measures on poultry farms, including strict protocols for hygiene, sanitation, and disease prevention. These protocols should encompass the segregation of potentially infected flocks, proper cleaning of farming equipment entering or exiting the facilities, and disinfection of contaminated areas with proper chemical or physical agents [93]. Simultaneously, suspected AIV cases should be immediately reported to local authorities. Countries such as Thailand, Malaysia, and Singapore have introduced their biosafety and biosecurity laws that eventually protect farmers and other authorities [94,95,96].

In addition, the updated post-2020 framework has included three distinct strategic plans tailored to the varying needs and circumstances of ASEAN member states. First, for countries currently free from avian influenza, the strategy focuses on preserving this status through extensive wildlife and cross-border surveillance, workforce development in diagnosis and epidemiology, enhanced early warning systems, increased public awareness, and strengthened regional collaboration. Next, countries that experience occasional outbreaks should focus on improving the capability and immediate control of existing outbreaks to recover avian influenza-free status. This includes early rapid detection, promoting disease controls at the border, strategic surveillance of poultry trading, increasing laboratory capacity (BSL3), enhancing public awareness, and the application of stricter legislation. Lastly, for countries with the sustained presence of avian influenza outbreaks, the focus is directed towards ensuring enough capacity to reduce the impact on the existing poultry industry as well as reducing the risk of human transmission. For this stage, some factors should be considered, which include collaboration between different stakeholders, prioritizing passive and active surveillance, introducing compensation schemes to the affected farmers, applying farm biosecurity, identifying AIV poultry production zones, implementing mandatory vaccination programs, and improving laboratory capacity [12,97]. These strategic plans provide a comprehensive and adaptable framework for ASEAN member states to effectively manage avian influenza based on their specific situations and needs.

## 5. Lesson Learned from Past Outbreaks

As a result of the contingency plans that have been implemented, management of the H5 HPAI and LPAI outbreaks that were reported throughout the ASEAN region has shown significant improvements. Participating states have introduced their local mitigation plans that include a holistic approach for the government, private sector, farmers, and/or community (Table 1). Over the past 10 years, enhanced strategic planning and risk management have been crucial in effectively controlling H5 outbreaks in several ASEAN nations.

The recent outbreak of HPAI H5N1 in Cambodia resulted in at least six human fatality cases from October 2023 to August 2024, emphasizing the critical need for effective disease control measures [104]. Learning from past events, the Cambodian CDC, together with local authorities, implemented a rapid response strategy following the national preparedness plan [98]. To prevent further human transmission, Cambodian authorities administered immediate antiviral treatment (Oseltamivir) to the identified close contacts [104]. Another strategy that has been implemented includes strengthening biosecurity controls in food quality and health safety [97]. This proactive approach, involving enhanced poultry chain management and zoning systems, particularly in hotspot areas, facilitated more effective containment of the virus, specifically at an early stage [92,105].

In contrast, Malaysia’s successful containment of the H5N1 outbreak in poultry farms in 2018 highlights the importance of early intervention and inter-agency collaboration. Following a self-declaration document to the World Organization for Animal Health (WOAH), the Malaysia Department of Veterinary Services, alongside a team of experts, implemented an emergency response to contain virus transmission [106]. This included strict quarantine measures, decontamination and containment of affected poultry farms, and active surveillance according to the Malaysia Manual for the Control of Highly Pathogenic Avian Influenza. In addition, public health interventions were also exercised to minimize human infection, which included movement restrictions in infected areas, alongside roadblocks by the Royal Malaysia Police around the zoning area. This resulted in the success of Malaysia eradicating the H5N1 outbreak within 43 days and regaining the HPAI H5N1 freedom status. However, a few issues during the HPAI control were identified, such as inadequate laboratory testing capabilities, insufficient government funding, and conflict with the affected farmers. Improvements are needed to prevent any recurrence of H5 outbreaks, especially with help at the community level.

While Cambodia and Malaysia provided valuable case studies, Laos offers another compelling example of effective H5 outbreak management. In the 2021 H5N6 outbreak, as a result of active AIV surveillance, the H5N6 infection was first detected in a 5-year-old child with close contact with ducks and chickens. The oropharyngeal and nasopharyngeal swab specimens tested positive for H5N6 AIV [107]. This approach is one of the strategies under the National Avian Influenza Control and Pandemic Preparedness Plan created by the Laos government with the United Nations (UN), which is to collect responses and perform disease surveillance such as influenza-like illness (ILI) and severe acute respiratory infections (SARI) surveillance [98]. Immediate investigation, including house-to-house interviews, was performed, and the collected data were used to detect any increases in the H5N6 case. This has resulted in the identification of the source of the outbreak as a poultry cluster within the child’s village, harboring the clade 2.3.4.4h HPAI H5N6. Control measures imposed by the Laos government to contain the outbreak encompassed a range of interventions: depopulation and decontamination of affected flocks, public awareness campaigns, enhanced biosecurity, and isolation of commercial poultry and wild birds. Therefore, based on the responses, measurement, and insights gained from this outbreak, the importance of sustaining continuous surveillance for ILI and SARI needs to be highlighted, especially in countries that have seasonal or consistent outbreaks. In addition, integrating artificial intelligence tools to enhance surveillance systems for wild birds could significantly benefit ASEAN nations, especially in predicting potential outbreaks and enabling more timely responses to emerging threats [108]. Ultimately, a coordinated response, including prompt action to contain outbreaks and a thorough investigation of the human–animal interface, remains vital for effective pandemic preparedness.

## 6. One Health Approaches

The interconnectedness of humans, agriculture, and wildlife in the ASEAN region, particularly through intensive poultry farming and live animal markets, creates an environment conducive to zoonotic disease emergence. Diverse ecosystems within ASEAN countries further facilitate viral reservoirs to thrive, with wild bird migration increasing the risk of H5 spread to humans and other animals [109,110]. This complex interplay underscores the One Health approach, which emphasizes a collaborative, cross-sectoral strategy to address the interconnectedness of human, animal, and environmental health [111]. The effectiveness of this approach has been proven in past successes against rabies, the Zika virus, and MERS-CoV, offering a promising framework for combating H5 infections.

One example that has proven the effectiveness of the One Health approach is Thailand’s response to the H5N1 outbreak in 2004. The government implemented an “X-ray survey” surveillance program that enables inter-ministerial collaboration to monitor signs of AIV infection in poultry, wild birds, and humans [112]. To mitigate the transboundary spread of H5N1, restrictions on the movement of poultry products in the affected regions were also implemented alongside heightened biosecurity measures in place. Extensive efforts were made through public awareness campaigns, which effectively encouraged the responsible reporting of sick poultry and discouraged the handling of dead birds [86]. These cross-sectoral interventions significantly reduced H5N1 cases in both humans and poultry, ultimately eradicating the outbreak in Thailand.

Other countries that were affected by the H5N1 outbreak in the early 2000s, such as Vietnam, also successfully implemented comprehensive control measures that followed the notion of the One Health approach. They developed an Integrated Operational Program for Avian and Human Influenza (OPI) framework to foster strong collaboration between ministries and organizations [113]. Since their first reported outbreak of H5N1, the Department of Animal Health (DAH), the National Center for Veterinary Diagnostics (NCVD), and provincial DAH laboratories have been monitoring influenza cases to identify and characterize H5N1 viruses in Vietnam. These efforts lead to the implementation of key ASEAN strategic goals such as depopulation of infected flocks and mass poultry vaccination. By 2005, Vietnam had substantially reduced H5N1 cases in both humans and poultry, and these remain low due to ongoing surveillance and interventions [114]. To date, Vietnamese authorities actively monitor live bird markets, integrating human, animal, and environmental surveillance to detect early signs of AIV outbreaks [115].

These events demonstrate that effectively addressing H5 infections requires a comprehensive One Health approach, integrating human, animal, and environmental health considerations. This approach ensures that all elements contributing to the viral spread are addressed through cross-sectoral collaboration among public health, agriculture, and environmental sectors, both nationally and internationally. This collaboration is critical to ensure timely information sharing, coordinated actions, and technical support, especially in the ASEAN region, where transboundary disease spread is a significant concern. However, pandemic preparedness necessitates a shift beyond short-term control measures towards sustainable prevention. This includes the establishment of ongoing biosecurity practices, the improvement of public health infrastructure, and the development of effective vaccine programs. With these preparations, it will enhance our capacity to mitigate H5 infections and prevent future outbreaks of avian influenza.

## 7. Conclusions

The agricultural landscape in ASEAN has evolved, with livestock farming now contributing 9.8% of the total GDP as of 2022 [116]. This sector plays a crucial role in food security and economic stability. However, the region faces challenges in controlling HPAI outbreaks. The dynamics of H5 are complex, influenced by factors such as viral virulence, stability, host susceptibility, mode of transmission, environmental conditions, and human interventions [117,118]. The ability of AIVs to undergo antigenic shift raises concerns about the emergence of novel HPAI H5 strains with pandemic potential [119], especially with the migration pattern of wild birds that have changed due to climate changes [120,121]. Therefore, a holistic approach is crucial to effectively address this complex issue.

Historically, the strategies that were implemented to combat H5 infections were mainly reactive, in which the governments addressed the outbreaks as they emerged. The initiatives were more focused on short-term containment measures, such as immediate culling and movement restrictions of poultry. These efforts often lacked the systematic cross-sectoral coordination that exists in the current strategies, where there is a stronger emphasis on integrating public health, animal health, and environmental management. The current approach, which is more proactive and preventative, aims to detect and control the virus before widespread outbreaks occur. Recently, programs such as vaccination, ongoing surveillance, and biosecurity practices have been implemented as measures for sustainable control and prevention of H5 infections, which address not only the immediate outbreak but also the underlying factors contributing to the virus’s spread. With increased international cooperation, countries in the ASEAN region are able to share data and align their responses to prevent the spread of H5 infections across borders. By learning from past experiences and utilizing modern advancements (artificial intelligence surveillance, alternative antivirals, and vaccines), ASEAN countries will be able to improve their capacity to manage and mitigate the impact of H5 infections, thereby safeguarding public health and economic stability.

## Figures and Tables

**Table 1:** Avian influenza preparedness and mitigation plans in ASEAN member states.

Country	Local Mitigation/Preparedness Plans(s)	Year Published/Revised	References
Brunei	Influenza Pandemic Preparedness—Recommendations for Workplaces and Business Continuity Plan	2008	- *
Cambodia	Cambodia National Comprehensive Avian and Human Influenza Plan	2007	[98]
Indonesia	National Strategic Plan for Avian Influenza Control and Pandemic Influenza Preparedness	2006	[98]
Laos	National Avian Influenza Control and Pandemic Preparedness Plan	2006	[98]
Malaysia	National Influenza Pandemic Preparedness Plan	2008	[99]
	Manual for the Control of Highly Pathogenic Avian Influenza	2005	[100]
Myanmar	National Strategic Plan for Prevention and Control of Avian Influenza and Human Influenza Pandemic Preparedness and Response	2006	[101]
Philippines	Avian Influenza Protection Program Manuals of Procedure (2020)	2020	[102]
Singapore	MOH Pandemic Readiness and Response Plan for Influenza and Other Acute Respiratory Diseases	2014	- *
Thailand	Thailand National Strategic Plan for Emerging Infectious Disease Preparedness, Prevention, and Response	2013	[98]
Vietnam	Vietnam Integrated Program on Avian Influenza, Pandemic Preparedness, and Emerging Infectious Diseases 2011–2015	2011	[103]

* Document has been retracted by the respective government.

## Data Availability

Not applicable.

## References

[1] Chan P.K.S. (2002). Outbreak of Avian Influenza A (H5N1) Virus Infection in Hong Kong in 1997. Clin. Infect. Dis..

[2] Coker R.J., Hunter B.M., Rudge J.W., Liverani M., Hanvoravongchai P. (2011). Emerging Infectious Diseases in Southeast Asia: Regional Challenges to Control. Lancet.

[3] Gutiérrez R.A., Naughtin M.J., Horm S.V., San S., Buchy P. (2009). A(H5N1) Virus Evolution in South East Asia. Viruses.

[4] AbuBakar U., Amrani L., Kamarulzaman F.A., Karsani S.A., Hassandarvish P., Khairat J.E. (2023). Avian Influenza Virus Tropism in Humans. Viruses.

[5] Caserta L.C., Frye E.A., Butt S.L., Laverack M., Nooruzzaman M., Covaleda L.M., Thompson A.C., Koscielny M.P., Cronk B., Johnson A. (2024). Spillover of Highly Pathogenic Avian Influenza H5N1 Virus to Dairy Cattle. Nature.

[6] Sreenivasan C.C., Li F., Wang D. (2024). Emerging Threats of Highly Pathogenic Avian Influenza A (H5N1) in US Dairy Cattle: Understanding Cross-Species Transmission Dynamics in Mammalian Hosts. Viruses.

[7] Du R., Cui Q., Chen Z., Zhao X., Lin X., Rong L. (2023). Revisiting Influenza A Virus Life Cycle from a Perspective of Genome Balance. Virol. Sin..

[8] Dou D., Revol R., Östbye H., Wang H., Daniels R. (2018). Influenza A Virus Cell Entry, Replication, Virion Assembly and Movement. Front. Immunol..

[9] Kosik I., Yewdell J.W. (2019). Influenza Hemagglutinin and Neuraminidase: Yin–Yang Proteins Coevolving to Thwart Immunity. Viruses.

[10] Wang Y., Song T., Li K., Jin Y., Yue J., Ren H., Liang L. (2019). Different Subtypes of Influenza Viruses Target Different Human Proteins and Pathways Leading to Different Pathogenic Phenotypes. Biomed Res. Int..

[11] U.S. Center for Disease Control and Prevention Avian Influenza (Bird Flu). https://www.cdc.gov/bird-flu/index.html.

[12] The Association of Southeast Asian Nations Post-2020 Avian Influenza Control Framework in ASEAN. https://asean.org/book/post-2020-avian-influenza-control-framework-in-asean/.

[13] Ammali N., Kara R., Guetarni D., Chebloune Y. (2024). Highly Pathogenic Avian Influenza H5N8 and H5N1 Outbreaks in Algerian Avian Livestock Production. Comp. Immunol. Microbiol. Infect. Dis..

[14] Zhu W., Li X., Dong J., Bo H., Liu J., Yang J., Zhang Y., Wei H., Huang W., Zhao X. (2022). Epidemiologic, Clinical, and Genetic Characteristics of Human Infections with Influenza A(H5N6) Viruses, China. Emerg. Infect. Dis..

[15] Burggraaf S., Karpala A.J., Bingham J., Lowther S., Selleck P., Kimpton W., Bean A.G.D. (2014). H5N1 Infection Causes Rapid Mortality and High Cytokine Levels in Chickens Compared to Ducks. Virus Res..

[16] Poovorawan Y., Pyungporn S., Prachayangprecha S., Makkoch J. (2013). Global Alert to Avian Influenza Virus Infection: From H5N1 to H7N9. Pathog. Glob. Health.

[17] Kintz E., Trzaska W.J., Pegg E., Perry W., Tucker A.W., Kyriakides A., Antic D., Callaghan K., Wilson A.J. (2024). The Risk of Acquiring Avian Influenza from Commercial Poultry Products and Hen Eggs: A Qualitative Assessment. Microb. Risk Anal..

[18] James J., Warren C.J., De Silva D., Lewis T., Grace K., Reid S.M., Falchieri M., Brown I.H., Banyard A.C. (2023). The Role of Airborne Particles in the Epidemiology of Clade 2.3.4.4b H5N1 High Pathogenicity Avian Influenza Virus in Commercial Poultry Production Units. Viruses.

[19] Gutiérrez R.A., Buchy P. (2012). Contaminated Soil and Transmission of Influenza Virus (H5N1). Emerg. Infect. Dis..

[20] Xu X., Subbarao K., Cox N.J., Guo Y. (1999). Genetic Characterization of the Pathogenic Influenza A/Goose/Guangdong/1/96 (H5N1) Virus: Similarity of Its Hemagglutinin Gene to Those of H5N1 Viruses from the 1997 Outbreaks in Hong Kong. Virology.

[21] Yee K.S., Carpenter T.E., Cardona C.J. (2009). Epidemiology of H5N1 Avian Influenza. Comp. Immunol. Microbiol. Infect. Dis..

[22] Charostad J., Rukerd M.R.Z., Mahmoudvand S., Bashash D., Hashemi S.M.A., Nakhaie M., Zandi K. (2023). A Comprehensive Review of Highly Pathogenic Avian Influenza (HPAI) H5N1: An Imminent Threat at Doorstep. Travel Med. Infect. Dis..

[23] Chen H., Smith G.J.D., Li K.S., Wang J., Fan X.H., Rayner J.M., Vijaykrishna D., Zhang J.X., Zhang L.J., Guo C.T. (2006). Establishment of Multiple Sublineages of H5N1 Influenza Virus in Asia: Implications for Pandemic Control. Proc. Natl. Acad. Sci. USA.

[24] Harvey J.A., Mullinax J.M., Runge M.C., Prosser D.J. (2023). The Changing Dynamics of Highly Pathogenic Avian Influenza H5N1: Next Steps for Management & Science in North America. Biol. Conserv..

[25] Nidra F.Y., Monir M.B., Dewan S.M.R. (2024). Avian Influenza A (H5N1) Outbreak 2024 in Cambodia: Worries Over the Possible Spread of the Virus to Other Asian Nations and the Strategic Outlook for Its Control. Environ. Health Insights.

[26] Peiris J.S.M., De Jong M.D., Guan Y. (2007). Avian Influenza Virus (H5N1): A Threat to Human Health. Clin. Microbiol. Rev..

[27] Mok C.K.P., Guan W.D., Liu X.Q., Lamers M.M., Li X.B., Wang M., Zhang T.J.S., Zhang Q.L., Li Z.T., Huang J.C. (2015). Genetic Characterization of Highly Pathogenic Avian Influenza A(H5N6) Virus, Guangdong, China. Emerg. Infect. Dis..

[28] Yang Y., Halloran M.E., Sugimoto J.D., Longini I.M. (2007). Detecting Human-to-Human Transmission of Avian Influenza A (H5N1). Emerg. Infect. Dis..

[29] Alexander D.J., Brown I.H. (2009). History of Highly Pathogenic Avian Influenza. Sci. Tech. Rev..

[30] Lee C.-C.D., Zhu H., Huang P.-Y., Peng L., Chang Y.-C., Yip C.-H., Li Y.-T., Cheung C.-L., Compans R., Yang C. (2014). Emergence and Evolution of Avian H5N2 Influenza Viruses in Chickens in Taiwan. J. Virol..

[31] Cheng M.C., Soda K., Lee M.S., Lee S.H., Sakoda Y., Kida H., Wang C.H. (2010). Isolation and Characterization of Potentially Pathogenic H5N2 Influenza Virus from a Chicken in Taiwan in 2008. Avian Dis..

[32] Okamatsu M., Saito T., Yamamoto Y., Mase M., Tsuduku S., Nakamura K., Tsukamoto K., Yamaguchi S. (2007). Low Pathogenicity H5N2 Avian Influenza Outbreak in Japan during the 2005-2006. Vet. Microbiol..

[33] Yamazaki Y., Doy M., Okabe N., Yasui Y., Nakashima K., Fujieda T., Yamato S.I., Kawata Y., Ogata T. (2009). Serological Survey of Avian H5N2-Subtype Influenza Virus Infections in Human Populations. Arch. Virol..

[34] Mahase E. (2024). Bird Flu: First Person with Confirmed H5N2 Infection Dies. BMJ.

[35] Adibah N.M., Zailina H., Arshad S.S. (2017). Avian Influenza Outbreaks in Malaysia, 1980-2017. Asia Pac. Environ. Occup. Health J..

[36] Lee J.H., Pascua P.N.Q., Song M.-S., Baek Y.H., Kim C.-J., Choi H.-W., Sung M.-H., Webby R.J., Webster R.G., Poo H. (2009). Isolation and Genetic Characterization of H5N2 Influenza Viruses from Pigs in Korea. J. Virol..

[37] Zhao G., Gu X., Lu X., Pan J., Duan Z., Zhao K., Gu M., Liu Q., He L., Chen J. (2012). Novel Reassortant Highly Pathogenic H5N2 Avian Influenza Viruses in Poultry in China. PLoS ONE.

[38] Baek Y.H., Pascua P.N.Q., Song M.S., Park K.J., Kwon H.I., Lee J.H., Kim S.Y., Moon H.J., Kim C.J., Choi Y.K. (2010). Surveillance and Characterization of Low Pathogenic H5 Avian Influenza Viruses Isolated from Wild Migratory Birds in Korea. Virus Res..

[39] Sultan S., Bui V.N., Hill N.J., Hussein I.T.M., Trinh D.Q., Inage K., Hashizume T., Runstadler J.A., Ogawa H., Imai K. (2016). Genetic Characterization of H5N2 Influenza Viruses Isolated from Wild Birds in Japan Suggests Multiple Reassortment. Arch. Virol..

[40] Guang-jian Z., Zong-shuai L., Yan-li Z., Shi-jin J., Zhi-jing X. (2012). Genetic Characterization of a Novel Influenza A Virus H5N2 Isolated from a Dog in China. Vet. Microbiol..

[41] Belot G., Claes F., Von Dobschuetz S., Kamata A., Newman S., Chanthavisouk C., Phommachanh P., Wongsathapornchai K., Fusheng G., Edwards J. (2014). Avian Influenza A(H5N6): The Latest Addition to Emerging Zoonotic Avian Influenza Threats in East and Southeast Asia. Empress Watch..

[42] Kang Y., Liu L., Feng M., Yuan R., Huang C., Tan Y., Gao P., Xiang D., Zhao X., Li Y. (2017). Highly Pathogenic H5N6 Influenza A Viruses Recovered from Wild Birds in Guangdong, Southern China, 2014-2015. Sci. Rep..

[43] He Z., Wang X., Lin Y., Feng S., Huang X., Zhao L., Zhang J., Ding Y., Li W., Yuan R. (2023). Genetic Characteristics of Waterfowl-Origin H5N6 Highly Pathogenic Avian Influenza Viruses and Their Pathogenesis in Ducks and Chickens. Front. Microbiol..

[44] Bi Y., Mei K., Shi W., Liu D., Yu X., Gao Z., Zhao L., Gao G.F., Chen J., Chen Q. (2015). Two Novel Reassortants of Avian Influenza A (H5N6) Virus in China. J. Gen. Virol..

[45] Pan M., Gao R., Lv Q., Huang S., Zhou Z., Yang L., Li X., Zhao X., Zou X., Tong W. (2016). Human Infection with a Novel, Highly Pathogenic Avian Influenza A (H5N6) Virus: Virological and Clinical Findings. J. Infect..

[46] Zhang J., Chen Y., Shan N., Wang X., Lin S., Ma K., Li B., Li H., Liao M., Qi W. (2020). Genetic Diversity, Phylogeography, and Evolutionary Dynamics of Highly Pathogenic Avian Influenza A (H5N6) Viruses. Virus Evol..

[47] Bean A.G.D., Baker M.L., Stewart C.R., Cowled C., Deffrasnes C., Wang L.F., Lowenthal J.W. (2013). Studying Immunity to Zoonotic Diseases in the Natural Host—Keeping It Real. Nat. Rev. Immunol..

[48] Wang B., Su Q., Luo J., Li M., Wu Q., Chang H., Du J., Huang C., Ma J., Han S. (2021). Differences in Highly Pathogenic H5N6 Avian Influenza Viral Pathogenicity and Inflammatory Response in Chickens and Ducks. Front. Microbiol..

[49] World Health Organization Surveillance in Emergencies. https://www.who.int/westernpacific/wpro-emergencies/surveillance.

[50] Sun Y., Hu Z., Zhang X., Chen M., Wang Z., Xu G., Bi Y., Tong Q., Wang M., Sun H. (2020). An R195K Mutation in the PA-X Protein Increases the Virulence and Transmission of Influenza A Virus in Mammalian Hosts. J. Virol..

[51] Herfst S., Mok C.K.P., van den Brand J.M.A., van der Vliet S., Rosu M.E., Spronken M.I., Yang Z., de Meulder D., Lexmond P., Bestebroer T.M. (2018). Human Clade 2.3.4.4 A/H5N6 Influenza Virus Lacks Mammalian Adaptation Markers and Does Not Transmit via the Airborne Route between Ferrets. mSphere.

[52] McParland P.J., Allan G.M., McCracken R.M., McNulty M.S. (1985). Isolation of a Highly Pathogenic Influenza Virus from Turkeys. Avian Pathol..

[53] Yamaguchi E., Hayama Y., Murato Y., Sawai K., Kondo S., Yamamoto T. (2024). A Case-Control Study of the Infection Risk of H5N8 Highly Pathogenic Avian Influenza in Japan during the Winter of 2020–2021. Res. Vet. Sci..

[54] Nagarajan S., Kumar M., Murugkar H.V., Tripathi S., Shukla S., Agarwal S., Dubey G., Nagi R.S., Singh V.P., Tosh C. (2017). Novel Reassortant Highly Pathogenic Avian Influenza (H5N8) Virus in Zoos, India. Emerg. Infect. Dis..

[55] Si Y.J., Jang S.-G., Kim Y.-I., Casel M.A.B., Kim D.-J., Ji H.Y., Choi J.H., Gil J.R., Rollon R., Jang H. (2024). Evolutional Dynamics of Highly Pathogenic Avian Influenza H5N8 Genotypes in Wintering Bird Habitats: Insights from South Korea’s 2020–2021 Season. One Health.

[56] Lee M.S., Chen L.H., Chen Y.P., Liu Y.P., Li W.C., Lin Y.L., Lee F. (2016). Highly Pathogenic Avian Influenza Viruses H5N2, H5N3, and H5N8 in Taiwan in 2015. Vet. Microbiol..

[57] Ku K.B., Park E.H., Yum J., Kim J.A., Oh S.K., Seo S.H. (2014). Highly Pathogenic Avian Influenza A(H5N8) Virus from Waterfowl, South Korea, 2014. Emerg. Infect. Dis..

[58] Li M., Liu H., Bi Y., Sun J., Wong G., Liu D., Li L., Liu J., Chen Q., Wang H. (2017). Highly Pathogenic Avian Influenza A(H5N8) Virus in Wild Migratory Birds, Qinghai Lake, China. Emerg. Infect. Dis..

[59] Caliendo V., Leijten L., Begeman L., Poen M.J., Fouchier R.A.M., Beerens N., Kuiken T. (2020). Enterotropism of Highly Pathogenic Avian Influenza Virus H5N8 from the 2016/2017 Epidemic in Some Wild Bird Species. Vet. Res..

[60] Floyd T., Banyard A.C., Lean F.Z., Byrne A.M., Fullick E., Whittard E., Mollett B.C., Bexton S., Swinson V., Macrelli M. (2021). Encephalitis and Death in Wild Mammals at a Rehabilitation Center after Infection with Highly Pathogenic Avian Influenza A(H5N8) Virus, United Kingdom. Emerg. Infect. Dis..

[61] Shin D.L., Siebert U., Lakemeyer J., Grilo M., Pawliczka I., Wu N.H., Valentin-Weigand P., Haas L., Herrler G. (2019). Highly Pathogenic Avian Influenza A(H5N8) Virus in Gray Seals, Baltic Sea. Emerg. Infect. Dis..

[62] Pyankova O.G., Susloparov I.M., Moiseeva A.A., Kolosova N.P., Onkhonova G.S., Danilenko A.V., Vakalova E.V., Shendo G.L., Nekeshina N.N., Noskova L.N. (2021). Isolation of Clade 2.3.4.4b A(H5N8), a Highly Pathogenic Avian Influenza Virus, from a Worker during an Outbreak on a Poultry Farm, Russia, December 2020. Eurosurveillance.

[63] Musa E., Nia Z.M., Bragazzi N.L., Leung D., Lee N., Kong J.D. (2024). Avian Influenza: Lessons from Past Outbreaks and an Inventory of Data Sources, Mathematical and AI Models, and Early Warning Systems for Forecasting and Hotspot Detection to Tackle Ongoing Outbreaks. Healthcare.

[64] Subedi D., Farhan M.H.R., Niraula A., Shrestha P., Chandran D., Acharya K.P., Ahmad M. (2024). Avian Influenza in Low and Middle-Income Countries (LMICs): Outbreaks, Vaccination Challenges and Economic Impact. Pak. Vet. J..

[65] McLeod A., Morgan N., Prakash A., Hinrichs J. Economic and Social Impacts of Avian Influenza. https://openknowledge.fao.org/items/7f8e1b13-f130-474f-9ea7-65ea1449ecf4.

[66] The World Bank Countering Global Shocks. https://documents.worldbank.org/en/publication/documents-reports/documentdetail/220041468245427647/countering-global-shocks.

[67] Pramuwidyatama M.G., Indrawan D., Boeters M., Poetri O.N., Saatkamp H.W., Hogeveen H. (2023). Economic Impact of Highly Pathogenic Avian Influenza Outbreaks in Western Java Smallholder Broiler Farms. Prev. Vet. Med..

[68] Elçi C. (2006). The Impact of HPAI of the H5N1 Strain on Economies of Affected Countries. International Conference on Human and Economic Resources.

[69] Hinrichs J., Sims L., Mcleod A. (2006). Some Direct Costs of Control for Avian Influenza. 11th International Symposium on Veterinary Epidemiology and Economics.

[70] Simmerman J.M., Lertiendumrong J., Dowell S.F., Uyeki T., Olsen S.J., Chittaganpitch M., Chunsutthiwat S., Tangcharoensathien V. (2006). The Cost of Influenza in Thailand. Vaccine.

[71] Malik Y.A. (2023). Impact of Influenza in South-East Asia. Int. J. Infect. Dis..

[72] Burgos S., Burgos S.A. (2007). Avian Influenza Outbreaks in Southeast Asia Affects Prices, Markets and Trade: A Short Case Study. Int. J. Poult. Sci..

[73] Buaprommee N., Polyorat K. (2016). The Antecedents of Purchase Intention of Meat with Traceability in Thai Consumers. Asia Pac. Manag. Rev..

[74] Vapnek J. Regulatory Measures Against Outbreaks of Highly Pathogenic Avian Influenza. https://www.fao.org/fileadmin/user_upload/legal/docs/lpo82.pdf.

[75] Azhar M., Lubis A.S., Siregar E.S., Alders R.G., Brum E., McGrane J., Morgan I., Roeder P. (2010). Participatory Disease Surveillance and Response in Indonesia: Strengthening Veterinary Services and Empowering Communities to Prevent and Control Highly Pathogenic Avian Influenza. Avian Dis..

[76] Leong H.K., Goh C.S., Chew S.T., Lim C.W., Lin Y.N., Chang S.F., Yap H.H., Chua S.B. (2008). Prevention and Control of Avian Influenza in Singapore. Ann. Acad. Med. Singap..

[77] Pribadi E.S., Hanun H., Haryanto A.P., Sutarman D.C., Utami S.S., Harahap R.H., Safika S., Kompudu A.J.M., Schoonman L., Utomo G.B. (2023). Correlation Strength Assessment of Animal Husbandry Components to the Implementation of ASEAN Good Animal Husbandry Practices: A Case Study in Layer Farming. J. Ilmu-Ilmu Peternak..

[78] Nielsen S.S., Alvarez J., Bicout D.J., Calistri P., Canali E., Drewe J.A., Garin-Bastuji B., Rojas J.L.G., Gortázar C., Herskin M. (2023). Vaccination of Poultry against Highly Pathogenic Avian Influenza—Part 1. Available Vaccines and Vaccination Strategies. EFSA J..

[79] Zeng X.Y., He X.W., Meng F., Ma Q., Wang Y., Bao H.M., Liu Y.J., Deng G.H., Shi J.Z., Li Y.B. (2022). Protective Efficacy of an H5/H7 Trivalent Inactivated Vaccine (H5-Re13, H5-Re14, and H7-Re4 Strains) in Chickens, Ducks, and Geese against Newly Detected H5N1, H5N6, H5N8, and H7N9 Viruses. J. Integr. Agric..

[80] Bertran K., Moresco K., Swayne D.E. (2015). Impact of Vaccination on Infection with Vietnam H5N1 High Pathogenicity Avian Influenza Virus in Hens and the Eggs They Lay. Vaccine.

[81] Mighell E., Ward M.P. (2021). African Swine Fever Spread across Asia, 2018–2019. Transbound. Emerg. Dis..

[82] Tibenderana J.R. (2023). Stamping out Marburg Virus Disease in Tanzania: Cutting-Edge Interventions and Recommendations for a Healthier Future. IJS Glob. Health.

[83] Looi L.M., Chua K.B. (2007). Lessons from the Nipah Virus Outbreak in Malaysia. Malays. J. Pathol..

[84] Norulhuda W., Makmal T.J., Kawasan V., Bharu K., Kerian J.K. (2018). An Overview of Highly Pathogenic Avian Influenza (H5N1) Outbreak Cases in Kelantan, West Malaysia in Year 2017. Malays. J. Vet. Res..

[85] Magalhães R.J.S., Pfeiffer D.U., Otte J. (2010). Evaluating the Control of HPAIV H5N1 in Vietnam: Virus Transmission within Infected Flocks Reported before and after Vaccination. BMC Vet. Res..

[86] Tiensin T., Chaitaweesub P., Songserm T., Chaisingh A., Hoonsuwan W., Buranathai C., Parakamawongsa T., Premashthira S., Amonsin A., Gilbert M. (2005). Highly Pathogenic Avian Influenza H5N1, Thailand, 2004. Emerg. Infect. Dis..

[87] Ziegler T., Moen A., Zhang W., Cox N.J. (2022). Global Influenza Surveillance and Response System: 70 Years of Responding to the Expected and Preparing for the Unexpected. Lancet.

[88] Mon H.H., Hadrill D., Brioudes A., Mon C.C.S., Sims L., Win H.H., Thein W.Z., Mok W.S., Kyin M.M., Maw M.T. (2021). Longitudinal Analysis of Influenza A(H5) Sero-Surveillance in Myanmar Ducks, 2006–2019. Microorganisms.

[89] Norrulashikin M.A., Yusof F., Hanafiah N.H.M., Norrulashikin S.M. (2021). Modelling Monthly Influenza Cases in Malaysia. PLoS ONE.

[90] Fournié G., Guitian J., Desvaux S., Cuong V.C., Dung D.H., Pfeiffer D.U., Mangtani P., Ghani A.C. (2013). Interventions for Avian Influenza A (H5N1) Risk Management in Live Bird Market Networks. Proc. Natl. Acad. Sci. USA.

[91] Zhou X., Wang Y., Liu H., Guo F., Doi S.A., Smith C., Clements A.C.A., Edwards J., Huang B., Magalhães R.J.S. (2018). Effectiveness of Market-Level Biosecurity at Reducing Exposure of Poultry and Humans to Avian Influenza: A Systematic Review and Meta-Analysis. J. Infect. Dis..

[92] Van Kerkhove M.D., Vong S., Guitian J., Holl D., Mangtani P., San S., Ghani A.C. (2009). Poultry Movement Networks in Cambodia: Implications for Surveillance and Control of Highly Pathogenic Avian Influenza (HPAI/H5N1). Vaccine.

[93] The Association of Southeast Asian Nations Asean Biosecurity Management Manual for Commercial Poultry Farming. https://asean.org/wp-content/uploads/2021/09/AMAF-33-Biosecurity-Manual.pdf.

[94] Osman N. (2018). The Legal Regulation of Biosafety Risk: A Comparative Legal Study with Singapore Biosafety Law. Ph.D. Thesis.

[95] Shafie N.F., Osman N.D. (2024). Overview of Biosecurity Legislation in Malaysia. Perdana: Int. J. Acad. Res..

[96] Soisangwan P. (2021). Biosafety and Biosecurity Law in Thailand: From Legislation to Practice. J. Biosaf. Biosecurity.

[97] The Association of Southeast Asian Nations Prevention, Control, and Eradication of Avian Influenza in ASEAN: Strategies and Success Stories. https://asean.org/wp-content/uploads/2012/07/HPAI-Strategies.pdf.

[98] Asia-Pacific Alliances for the Control of Influenza Pandemic Preparedness Plans for the Asia-Pacific Region. https://apaci.asia/influenza/pandemic-preparedness/pandemic-preparedness-plans-for-the-asia-pacific-region/.

[99] Ministry of Health Malaysia MYCDCGP—National Influenza Preparedness Plan. https://books.google.com.my/books?id=ncpcDwAAQBAJ.

[100] Department of Veterinary Services Malaysia Manual for the Control of Highly Pathogenic Avian Influenza (HPAI). https://www.woah.org/fileadmin/database/ASIA/Malaysia/Manual_for_the_control_of_Highly_Pathogenic_Avian_Influenza_(HPAI)_Malaysia.pdf.

[101] Ministry of Health Union of Myanmar National Strategic Plan for Prevention and Control of Avian Influenza and Human Influenza Pandemic Preparedness and Response. https://www.mohs.gov.mm/ckfinder/connector?command=Proxy&lang=en&type=Main&currentFolder=/Publications/CEU/CEU_May+2018/&hash=a6a1c319429b7abc0a8e21dc137ab33930842cf5&fileName=Pandemic+plan+(+latest)27-6-06+Eng.pdf.

[102] Department of Agriculture-Bureau of Animal Industry Philippines Avian Influenza Protection Programme Manuals of Procedure. https://drive.google.com/file/d/1YGacdiinAgXOOz0ZzHbxYygVws0Ys70S/view?pli=1.

[103] Ministry of Health Vietnam Vietnam Integrated National Operational Program on Avian Influenza, Pandemic Preparedness and Emerging Infectious Diseases (AIPED) 2011–2015. https://onehealth.org.vn/upload/upload/AIPED+2011-2015+-+Final.pdf.

[104] World Health Organization Avian Influenza A (H5N1)-Cambodia. https://www.who.int/emergencies/disease-outbreak-news/item/2024-DON501.

[105] Knobler S.L., Mack A., Mahmoud A., Lemon S.M., Institute of Medicine (2005). The Threat of Pandemic Influenza: Are We Ready. Workshop Summary.

[106] Hassan Nizam Q.N. Self-Declaration on the Recovery of Freedom from Highly Pathogenic Avian Influenza by Malaysia. http://www.dvs.gov.my/index.php/pages/view/538.

[107] Sengkeopraseuth B., Co K.C., Leuangvilay P., Mott J.A., Khomgsamphanh B., Somoulay V., Tsuyuoka R., Chiew M., Ketmayoon P., Jones J. (2022). First Human Infection of Avian Influenza A(H5N6) Virus Reported in Lao People’s Democratic Republic, February–March 2021. Influenza Other Respir. Viruses.

[108] Walsh D.P., Ma T.F., Ip H.S., Zhu J. (2019). Artificial Intelligence and Avian Influenza: Using Machine Learning to Enhance Active Surveillance for Avian Influenza Viruses. Transbound. Emerg. Dis..

[109] Blagodatski A., Trutneva K., Glazova O., Mityaeva O., Shevkova L., Kegeles E., Onyanov N., Fede K., Maznina A., Khavina E. (2021). Avian Influenza in Wild Birds and Poultry: Dissemination Pathways, Monitoring Methods, and Virus Ecology. Pathogens.

[110] Fair J.M., Al-Hmoud N., Alrwashdeh M., Bartlow A.W., Balkhamishvili S., Daraselia I., Elshoff A., Fakhouri L., Javakhishvili Z., Khoury F. (2024). Transboundary Determinants of Avian Zoonotic Infectious Diseases: Challenges for Strengthening Research Capacity and Connecting Surveillance Networks. Front. Microbiol..

[111] Horefti E. (2023). The Importance of the One Health Concept in Combating Zoonoses. Pathogens.

[112] Gilbert M., Chaitaweesub P., Parakamawongsa T., Premashthira S., Tiensin T., Kalpravidh W., Wagner H., Slingenbergh J. (2006). Free-Grazing Ducks and Highly Pathogenic Avian Influenza, Thailand. Emerg. Infect. Dis..

[113] Nguyen-Viet H., Lam S., Nguyen-Mai H., Trang D.T., Phuong V.T., Tuan N.D.A., Tan D.Q., Thuy N.T., Linh D.T., Pham-Duc P. (2022). Decades of Emerging Infectious Disease, Food Safety, and Antimicrobial Resistance Response in Vietnam: The Role of One Health. One Health.

[114] Creanga A., Nguyen D.T., Gerloff N., Do H.T., Balish A., Nguyen H.D., Jang Y., Dam V.T., Thor S., Jones J. (2013). Emergence of Multiple Clade 2.3.2.1 Influenza A (H5N1) Virus Subgroups in Vietnam and Detection of Novel Reassortants. Virology.

[115] Dao D.T., Coleman K.K., Bui V.N., Bui A.N., Tran L.H., Nguyen Q.D., Than S., Pulscher L.A., Marushchak L.V., Robie E.R. (2024). High Prevalence of Highly Pathogenic Avian Influenza: A Virus in Vietnam’s Live Bird Markets. Open Forum Infect. Dis..

[116] The Association of Southeast Asian Nations ASEAN Statistical Yearbook 2023. https://asean.org/wp-content/uploads/2023/12/ASEAN-Statistical-Yearbook-2023.pdf.

[117] Lee C.-Y. (2024). Exploring Potential Intermediates in the Cross-Species Transmission of Influenza A Virus to Humans. Viruses.

[118] Liang Y. (2023). Pathogenicity and Virulence of Influenza. Virulence.

[119] Huang P., Sun L., Li J., Wu Q., Rezaei N., Jiang S., Pan C. (2023). Potential Cross-Species Transmission of Highly Pathogenic Avian Influenza H5 Subtype (HPAI H5) Viruses to Humans Calls for the Development of H5-Specific and Universal Influenza Vaccines. Cell Discov..

[120] Sellwood C., Asgari-Jirhandeh N., Salimee S. (2007). Bird Flu: If or When? Planning for the next Pandemic. Postgrad. Med. J..

[121] Seri N.A., Rahman A.A. (2021). Impact of Climate Change on Migratory Birds in Asia. Pertanika J. Sci. Technol..

